# An improved statistical iterative algorithm for sparse-view and limited-angle CT image reconstruction

**DOI:** 10.1038/s41598-017-11222-z

**Published:** 2017-09-06

**Authors:** Zhanli Hu, Juan Gao, Na Zhang, Yongfeng Yang, Xin Liu, Hairong Zheng, Dong Liang

**Affiliations:** Lauterbur Research Center for Biomedical Imaging, Shenzhen Institutes of Advanced Technology, Chinese Academy of Sciences, Shenzhen, 518055 China

## Abstract

Because radiation is harmful to patients, it is important to reduce X-ray exposure in the clinic. For CT, reconstructions from sparse views or limited angle tomography are being used more frequently for low dose imaging. However, insufficient sampling data causes severe streak artifacts in images reconstructed using conventional methods. To solve this issue, various methods have recently been developed. In this paper, we improve a statistical iterative algorithm based on the minimization of the image total variation (TV) for sparse or limited projection views during CT image reconstruction. Considering the statistical nature of the projection data, the TV is performed under a penalized weighted least-squares (PWLS-TV) criterion. During implementation of the proposed method, the image reconstructed using the filtered back-projection (FBP) method is used as the initial value of the first iteration. Next, the feature refinement (FR) step is performed after each PWLS-TV iteration to extract the fine features lost in the TV minimization, which we refer to as ‘PWLS-TV-FR’.

## Introduction

Although the clinical value of CT is unquestionable, radiation exposure is harmful to patients^1, 2^. To date, many hardware-based scanning protocols have been proposed to reduce the CT scan radiation dose^3, 4^. Many software-based image reconstruction techniques have also been used to handle low-dose data from available CT scanners lacking hardware modifications^5–7^. In the CT field, the radiation dose is usually reduced by lowering the X-ray tube current, lowering the X-ray tube voltage, or sampling fewer views or using limited-angle projection. However, lowering X-ray exposure or using sparse or limited views increases noise and data inconsistency. Without adequate processing, it is difficult to reconstruct high-quality diagnostic CT images using conventional algorithms. Generally, a traditional CT algorithm using insufficient data will lead to degraded reconstruction accuracy and severe image artifacts^8^. Exact reconstruction methods with noise suppression capabilities intended to support dose reduction have been widely investigated.

One of the main strategies for low-dose CT image reconstruction restores the line integrals from acquired low-dose projection data, and a transform-based method has been presented to address the noise properties of low-mAs CT sinogram data. Li *et al*. determined a nonlinear relationship between the variance and the mean of acquired low-mAs sinogram data^9^, which facilitates low-dose CT image reconstruction. Based on this relationship, a framework for image reconstruction using low-mAs sinogram data with penalized re-weighted least squares was investigated by Wang *et al*.^10^. A general series of sophisticated CT image reconstruction methods were reported by Fessler *et al*.^11^.

Reducing projection views or limiting the scan angle is another strategy that has been used for CT dose reduction, as reducing the number of projections will clearly reduce the radiation dose^5^. In this study, we focused on CT image reconstruction from sparse-view or limited-angle projection data. Many iterative algorithms have been used for CT image reconstruction, and the actual iterative algorithms are well established^12^. Among them, statistical iterative reconstruction (SIR), which is used to model measurement statistics and imaging geometry, can significantly maintain the image quality in cases of insufficient sampling data, especially compared with the filtered back-projection (FBP) reconstruction algorithm^13^. The cost function of SIR is usually composed of a data-fidelity term and a regularization term. The data-fidelity term models the measurement statistics to ensure successful SIR image reconstruction, and the regularization term shows the prior information for the desired image intended to regularize the solution^14^. The traditional regularization term usually produces unfavorable smoothing effects on edge regions. To address this issue, several edge-preserving regularization terms have been presented, such as total variation (TV) regularization with a piecewise constant assumption (PCA)^15–17^. Numerous investigations have shown that high-quality CT images can be reconstructed via TV minimization from sparse-view sampling data. However, PCA often produces noticeable patchy artifacts in reconstructed images^5^.

Recently, we developed a statistical interior tomography approach for CT reconstruction^18^. Its potential usefulness have been demonstrated for feature preservation of interior tomography with truncated projection measurements. However, this algorithm is a little bit complex, involving FBP with projection extension, PWLS-TV and feature refinement and the last component is not very well characterized with the CT interior tomography application. Moreover, the algorithm was applied to a scanning configuration which is not practically used (namely the interior problem).

In this paper, we extend this method to a more practical protocol, sparse-view and limited-angle image reconstruction. Furthermore, the FBP algorithm is adopted instead of the FBP-EP algorithm which is time-consuming. To remove the patchy artifacts from the TV-based methods, we improved a statistical iterative image reconstruction algorithm based on minimizing the image TV that is specifically performed using PWLS criteria (PWLS-TV), which models the statistical properties of the projection data. Furthermore, after each PWLS-TV iteration, a feature refinement step (FR) is performed to restore the desired structure details that are lost in the TV minimization. For simplicity, the presented method is termed “PWLS-TV-FR”. The proposed PWLS-TV-FR method tends to relieve the PCA requirement by statistical modeling and iterative feature refinement.

The remainder of the paper is organized as follows. Section II briefly reviews the CT imaging model and describes the TV-regularized PWLS criterion in the image domain and the FR step. The experimental setup and evaluation metrics are also presented in this section. In section III and IV, the experimental results on a digital phantom and using micro-CT are reported, followed by a conclusion section.

## Methods

### PWLS-TV minimization

Based on the statistical properties of the projection data, the iterative PWLS approach including a data-fidelity term and a penalty term has been applied to the image domain for dual-energy X-ray CT reconstruction^19–21^. But the PWLS method for low-dose X-ray CT reconstruction are flawed in the suppression of noise and artifacts. According to our previous study, the TV-regularized PWLS algorithm we referred to as “PWLS-TV” can address this issue, which can be solved using the steepest descent algorithm. Based on the image gradient magnitude sparseness^22^, TV of the image is one of the most commonly used techniques:

1 $${\rm{TV}}({\rm{\mu }})=\sum _{s,t}\sqrt{{({\mu }_{s,t}-{\mu }_{s-1,t})}^{2}+{({\mu }_{s,t}-{\mu }_{s,t-1})}^{2}+\alpha }$$

in which *s* and *t* are the index of the location of the attenuation coefficients of the desired image and α is a small constant used for keeping the equation differentiable with respect to image intensity, which is assigned a value of ${10}^{-8}$ in this work.

Thus, given the current estimated image *μ* ^*n*^, a new image *μ* ^*n*+1^ can be calculated by minimizing the cost function of PWLS-TV, which can be described as follows:

2 $${\mu }^{n+1}={\mu }^{n}-{\tau }^{n}\times ({G}^{T}({{\rm{\Sigma }}}^{-1}(y-G{\mu }^{n})))-\beta \times \nabla TV({\mu }^{n})$$

in which ∇TV(μ^n^) denotes the gradient of TV(μ^n^), τ^n^ represents the gradient step size and *β* is a smoothing parameter balancing the degree of agreement between the estimated and the measured data. This can be computed as follows:

3 $${\tau }^{n}=\frac{{H}^{T}H}{{(GH)}^{T}({{\rm{\Sigma }}}^{-1}(GH))}\,with\,H\triangleq {G}^{T}({\Sigma }^{-1}(G{\mu }^{n}-y)).$$

### Feature refinement (FR)

**Figure 1:**
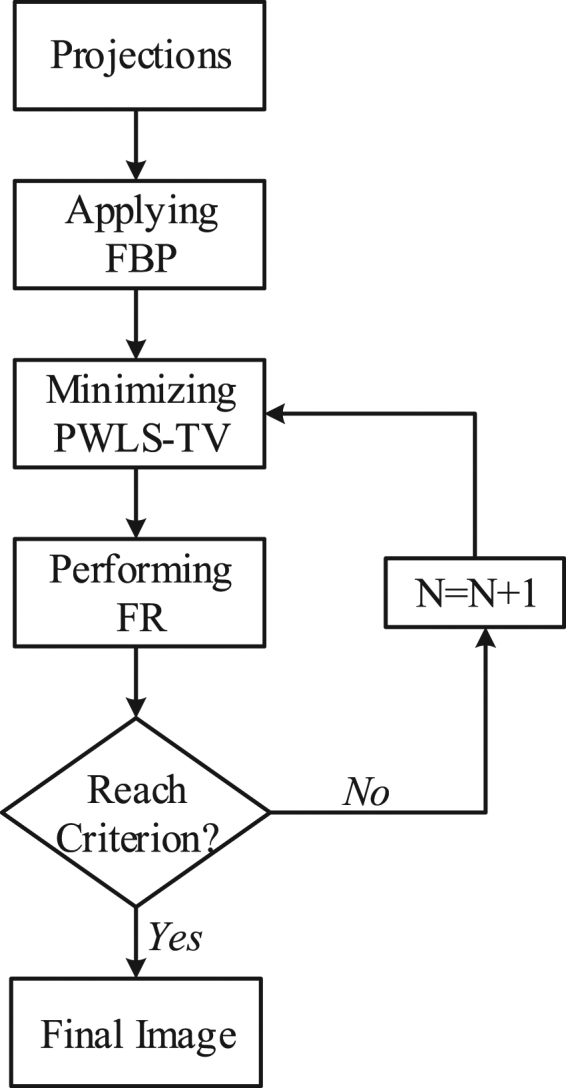
A flowchart of the proposed PWLS-TV-FR approach.

High-diagnostic CT images reconstructed from limited-angle or sparse-view acquisitions are very important in clinical practice. However, due to the piecewise constant assumption, some details in the reconstruction using the TV minimization are likely to be lost; in particular, the residual image between two successive iterations in PWLS-TV minimization might contain useful structural information, especially when the dose is very low or the projection data are insufficient. Motivated by this discovery, an FR was performed after each PWLS-TV minimization iteration to extract the main features of the loss from the residual image. This is described in the following equation:

4 $${\mu }_{new}^{n+1}=P[{\mu }^{n+1}+{f}_{t}^{n+1}\otimes ({\mu }^{n}-{\mu }^{n+1})]$$

in which the symbol ⊗ denotes point-wise multiplication, P is a non-negative control notation described as $${\rm{P}}[x]=\{\begin{array}{c}0,\,x < 0\\ x,\,x\ge 0\end{array}$$. ${f}_{t}^{n+1}\,\,$denotes a feature descriptor which is defined as:

5 $${f}_{t}^{n+1}=1-|\frac{2{\sigma }_{qp}+C}{{\sigma }_{p}^{2}+{\sigma }_{q}^{2}+C}|$$

in which the constants C is introduced for numerical stability. Local statistics σ_p_, σ_q_ and σ_pq_ at pixel *i* are defined as follows:

6 $${\sigma }_{p}({\rm{i}})={(\frac{1}{N-1}\sum _{i\in {p}_{i}}{({\mu }^{n+1}(i)-P(i))}^{2})}^{1/2}$$

7 $${\sigma }_{q}({\rm{i}})={(\frac{1}{N-1}\sum _{i\in {q}_{i}}{({\mu }_{d}(i)-Q(i))}^{2})}^{1/2}$$

8 $${\sigma }_{qp}({\rm{i}})=\frac{1}{N-1}\sum _{i\in ({p}_{i},{q}_{i})}({\mu }^{n+1}(i)-P(i))({\mu }_{d}(i)-Q(i))$$

in which ${\rm{P}}({\rm{i}})=\frac{1}{N}\sum _{i\in {p}_{i}}\mu (i)$, ${\rm{Q}}({\rm{i}})=\frac{1}{N}\sum _{i\in {q}_{i}}{\mu }_{d}(i)$, p_i_ and q_i_ denote two local image patches with a size of $\sqrt{N}\times \sqrt{N}$ centered at pixel *i* and extracted from ${\mu }^{n+1}$ and a degraded image ${\mu }_{d}$ obtained by a linear Gaussian filter from ${\mu }^{n+1}$, respectively. The nature of the presented model structure descriptor is a structure-related map and includes a contrast variation component and a structure correlation component. The former calculates the reduction of contrast variation due to the degraded operation, and the latter quantifies the structural correction between the original and degraded images^23^. The value of each element in the feature descriptor image f_t_ is in the interval [0, 1], and the larger the value, the more likely it belongs to part of the structure. In this paper, the proposed statistical iterative CT image reconstruction algorithm using the FR approach is referred to as “PWLS-TV-FR” and includes the three main steps shown in Fig. 1.

### Animal preparation and anesthesia administration

All animal experiments described in this work were approved by the Institutional Ethical Committee of Animal Experimentation of Shenzhen Institutes of Advanced Technology (Chinese Academy of Sciences). The experiments (Certificate number: SIAT-IRB-150203-YGS-QWB-A0088) were carried out strictly in accordance with governmental and international guidelines on animal experimentation. All efforts were made to minimize the usage amount of animals and the suffering during experiments according to the request of Biosafety and Animal Ethics. The mouse was anesthetized by intraperitoneal injection of ketamine (70 mg/kg) and xylazine (7 mg/kg) cocktail during CT scanning.

### Data availability

The datasets generated or analyzed during the current study are available from the corresponding author on reasonable request.

## Experiments

To validate the performance of PWLS-TV-FR, we performed a digital simulation experiment and a physical experiment. We used digital phantom simulation data that spanned 120 views to verify the effect of PWLS-TV-FR on sparse-view data. Additionally, sparse-view anesthetized mouse data and limited-angle mouse bone sample data were acquired from a commercial CT scanner to demonstrate the feasibility of the proposed method in practical applications for sparse-view or limited-angle sampling data. For both phantom simulation and Micro-CT data, Table 1 shows the idea projection number needed for FBP and the projection number used for proposed method.

**Table 1:** Projection number.

Projection number	Simulated data	Micro-CT data
Idea projection number of FBP	1160	450
Used projection number of PWLS-TV-FR	120	225

### Digital phantom simulations

**Figure 2:**
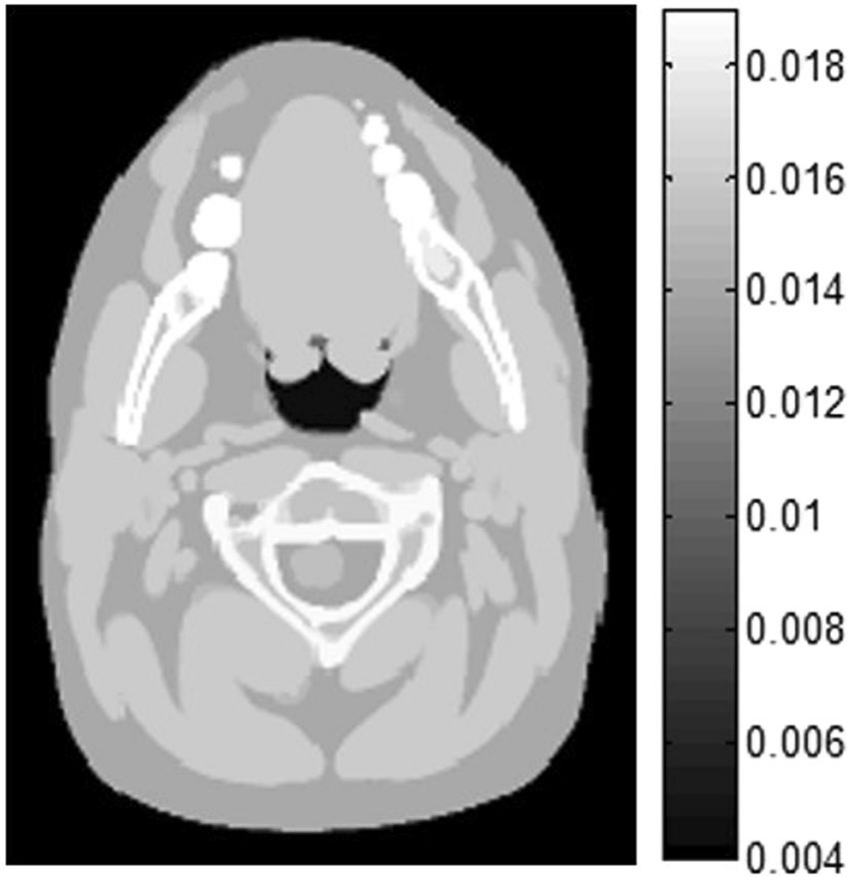
The head model for XCAT. Unless otherwise noted, all of the phantom images are shown in the same window [0.004 0.019].

A head model of the digital XCAT phantom was chosen as experimental simulation data (as shown in Fig. 2) to evaluate the performance of the FR step after each PWLS-TV iteration. To simulate sparse sampling, only 120 views were spanned on a circular orbit of 360°, and the number of bins per view was 672. The distance from the X-ray source to the detector was 1040 mm, and the distance from the rotation center to the curved detector was 570 mm.

### Micro-CT data

In this experiment, two scans were performed using a Micro-CT Skyscan1076 scanner with a normal-dose (70 kVp, 225 mAs) protocol. One scan was performed on an anesthetized mouse, in which 450 projections were uniformly collected over a 360° range, and another scan was performed on a mouse bone sample, in which 291 projections were uniformly collected over a 197.88° range, which can be considered limited-angle sampling. In this work, 450 projections of the anesthetized mouse were encoded from 0 to 449, and only even-numbered projections were selected for use as under-sampled data for a final total of 225 projections. Only the central slice projection was extracted, which is typical for fan-beam geometry. For each projection, 4000 detector elements were equally angularly distributed with a total length of 50.12 mm. The distance from the object to the curved detector was 165 mm, and the distance from the object to the X-ray source was 121 mm.

### Performance evaluation

(1) In this work, the root mean squared error (RMSE) was used to evaluate reconstruction accuracy and is described as:

9 $${\rm{RMSE}}=\sqrt{\frac{{\sum }_{m=1}^{Q}{(\mu (m)-{\mu }_{true}(m))}^{2}}{Q}}$$

in which $\mu$ denotes the reconstructed image, ${\mu }_{true}$ denotes the true image, m indexes the pixels of the image and Q is the total number of pixels in the image.

(2) To evaluate the noise reduction capability of different reconstruction algorithms, the peak signal to noise ratio (PSNR) metric was adopted in this paper and is as follows:

10 $${\rm{PSNR}}=10lo{g}_{10}[\frac{MA{X}^{2}({\mu }_{true})}{{\sum }_{m=1}^{Q}{(\mu (m)-{\mu }_{true}(m))}^{2}/(Q-1)}]$$

in which $\mu$ denotes the reconstructed image, ${\mu }_{true}$ denotes the true image, and ${\rm{MAX}}({\mu }_{true})$ represents the maximum intensity value of ${\mu }_{true}$.

## Results

### Digital phantom result

**Figure 3:**
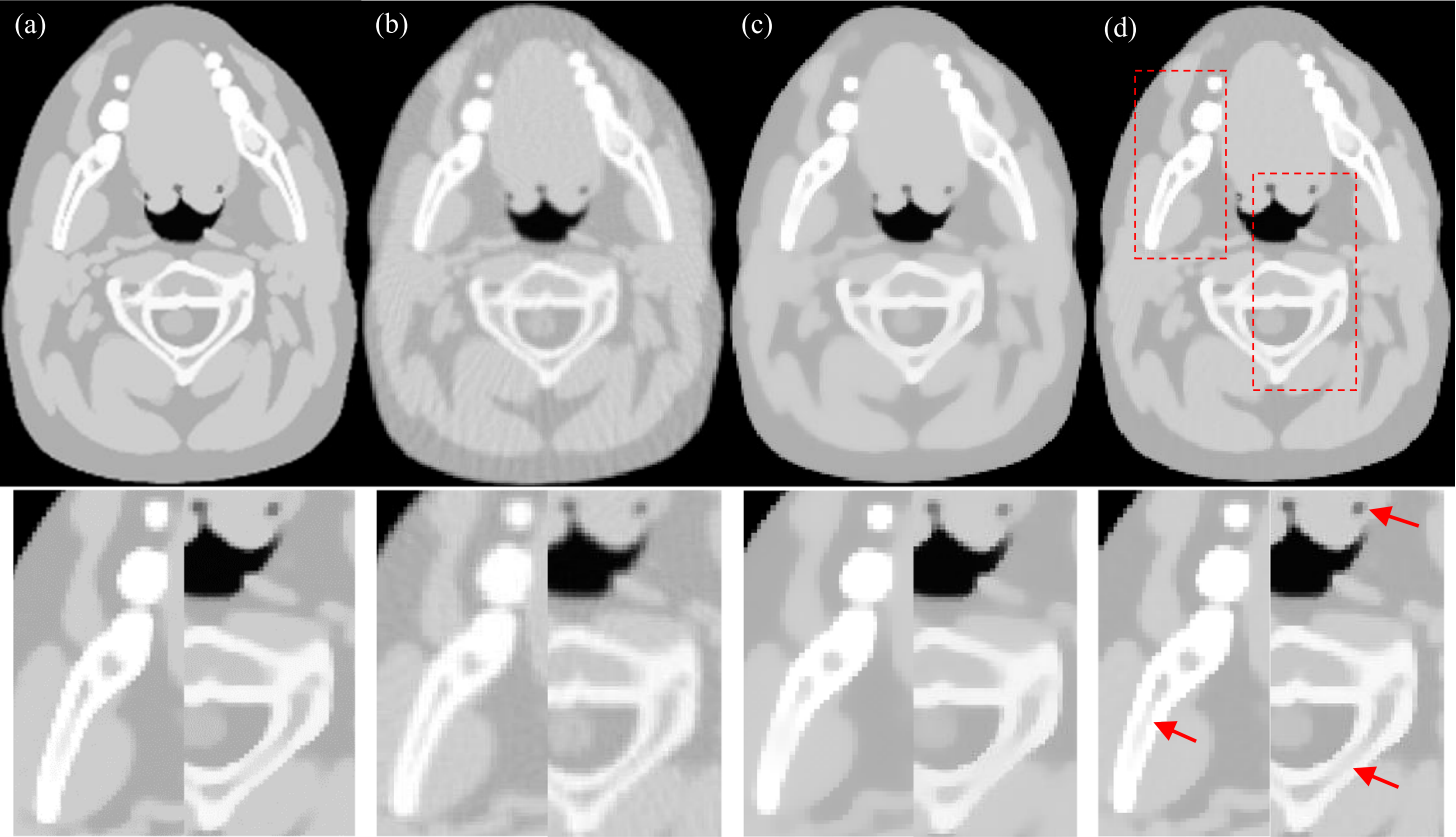
Reconstructed image of the head model from 120 views using different methods. The images in the second row are partially enlarged views of the first row. (**a**) Reference (**b**) FBP (**c**) PWLS-TV (**d**) PWLS-TV-FR.

**Figure 4:**
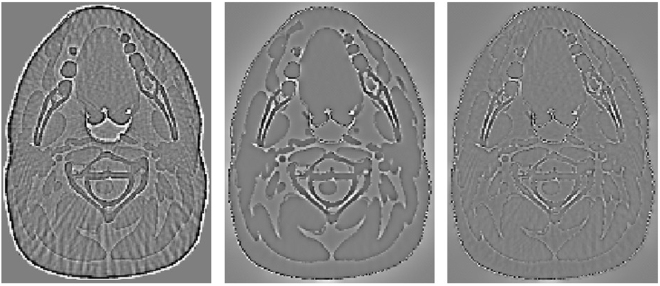
The residual image of the reconstructed results from Fig. 2. From left to right, the images are the residuals of results reconstructed by FBP, PWLS-TV and PWLS-TV-FR. The images are displayed in the window [−0.0015 0.0015].

In this experiment, with sparse sampling data, images were reconstructed using three methods, FBP, PWLS-TV and PWLS-TV-FR, which are shown with partial magnification in Fig. 3. From Fig. 3, it is clear that there are severe streak artifacts in the image reconstructed using FBP, whereas the PWLS-TV results exhibit fewer artifacts but degraded details. Moreover, the image reconstructed using PWLS-TV-FR showed noticeable improvements over that reconstructed using PWLS-TV by preserving small-scale and low-contrast structures, as indicated by the arrows in the amplified areas. Figure 4 shows residual reconstructed images (shown in Fig. 3) resulting from the true image subtracted from the reconstructed image. The smaller the features of the residual image, the more accurate the reconstructed image. As is clear from Fig. 4, the residual reconstructed image using PWLS-TV-FR has the smallest features, which indicates that the PWLS-TV-FR method for sparse-view data retains more details than either the PWLS-TV or FBP methods.

**Figure 5:**
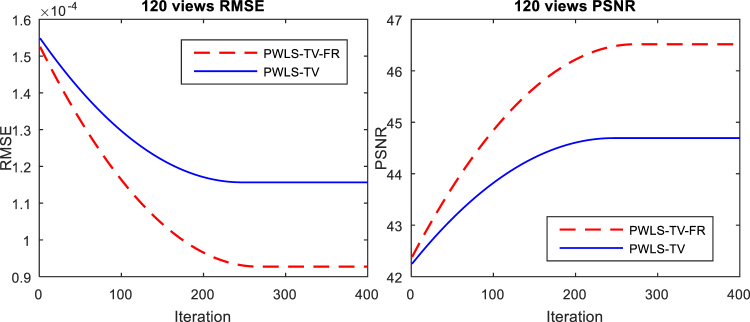
RMSE and PSNR as a function of the total number of image updates. The RMSE function is shown on the left, and the PSNR function is shown on the right.

**Figure 6:**
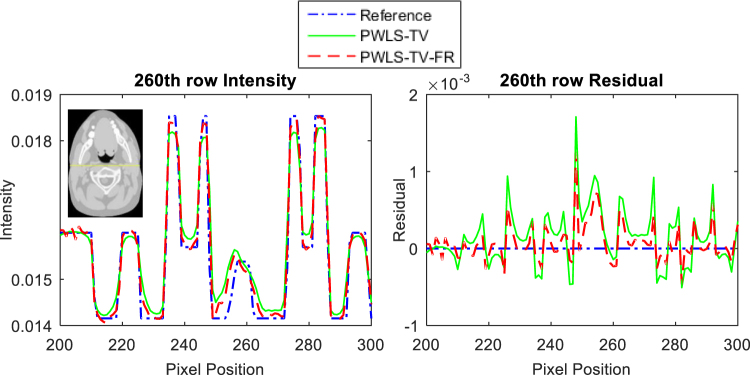
The left graph is profiles located at the pixel positions x from 200 to 300 and y = 260 of different results in Fig. 3. The graph on the right is the residual value corresponding to the left graph.

**Figure 7:**
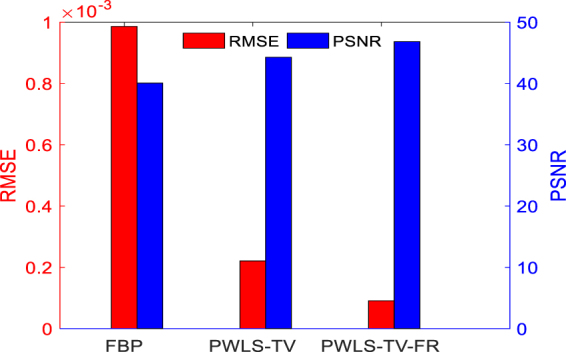
Image quality metrics.

In the process of updating images using the PWLS-TV or PWLS-TV-FR functions, the trend is that the image gradually becomes clearer and eventually reaches an extreme value as the number of iterations increases. However, as shown Fig. 5, whether it is evaluated by RMSE or by PSNR, the PWLS-TV-FR method always performs better than PWLS-TV. Figure 6 depicts the horizontal profiles of the reconstructions in Fig. 3 and the corresponding horizontal residual value. The result reconstructed using PWLS-TV-FR was closer to the reference profiles, and the residual values reconstructed using PWLS-TV-FR are much smaller than those using PWLS-TV; it is obvious that PWLS-TV-FR achieves better performance. Figure 7 shows a histogram of the RMSE and PSNR evaluation results for the reconstructions in Fig. 3. The red column represents the RMSE value, and the blue column represents the PSNR value. It is important that the RMSE value of PWLS-TV-FR is the minimum and the PSNR value of PWLS-TV-FR is the maximum, which verifies the validity of the PWLS-TV-FR algorithm for sparse-view digital phantom data.

### Micro-CT result

**Figure 8:**
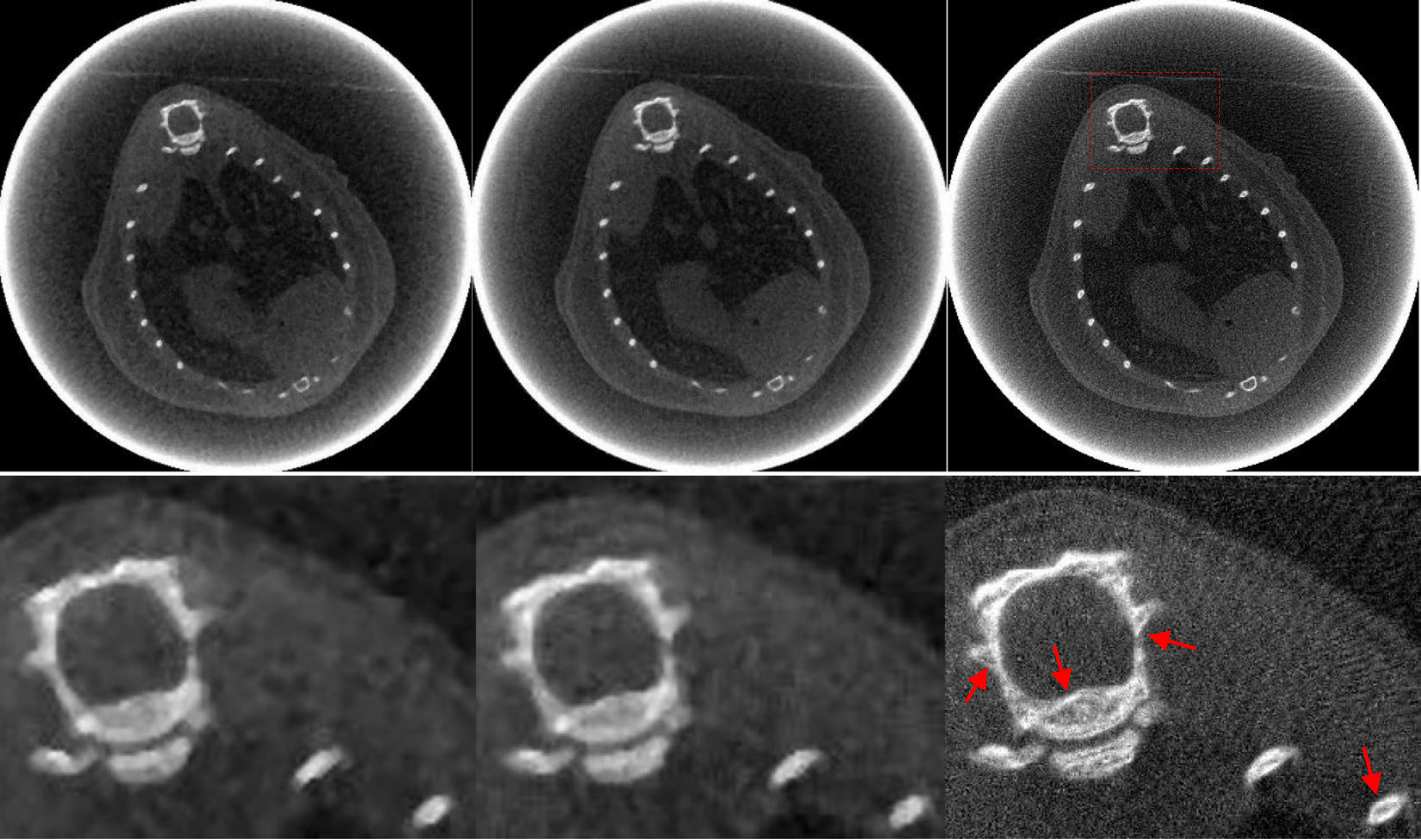
The anesthetized mouse data reconstructions from 225 views by different methods. From left to right, the images of the first row are the results obtained using FBP, PWLS-TV and PWLS-TV-FR method. The images of the second row are the partial enlarged view of the first row.

**Figure 9:**
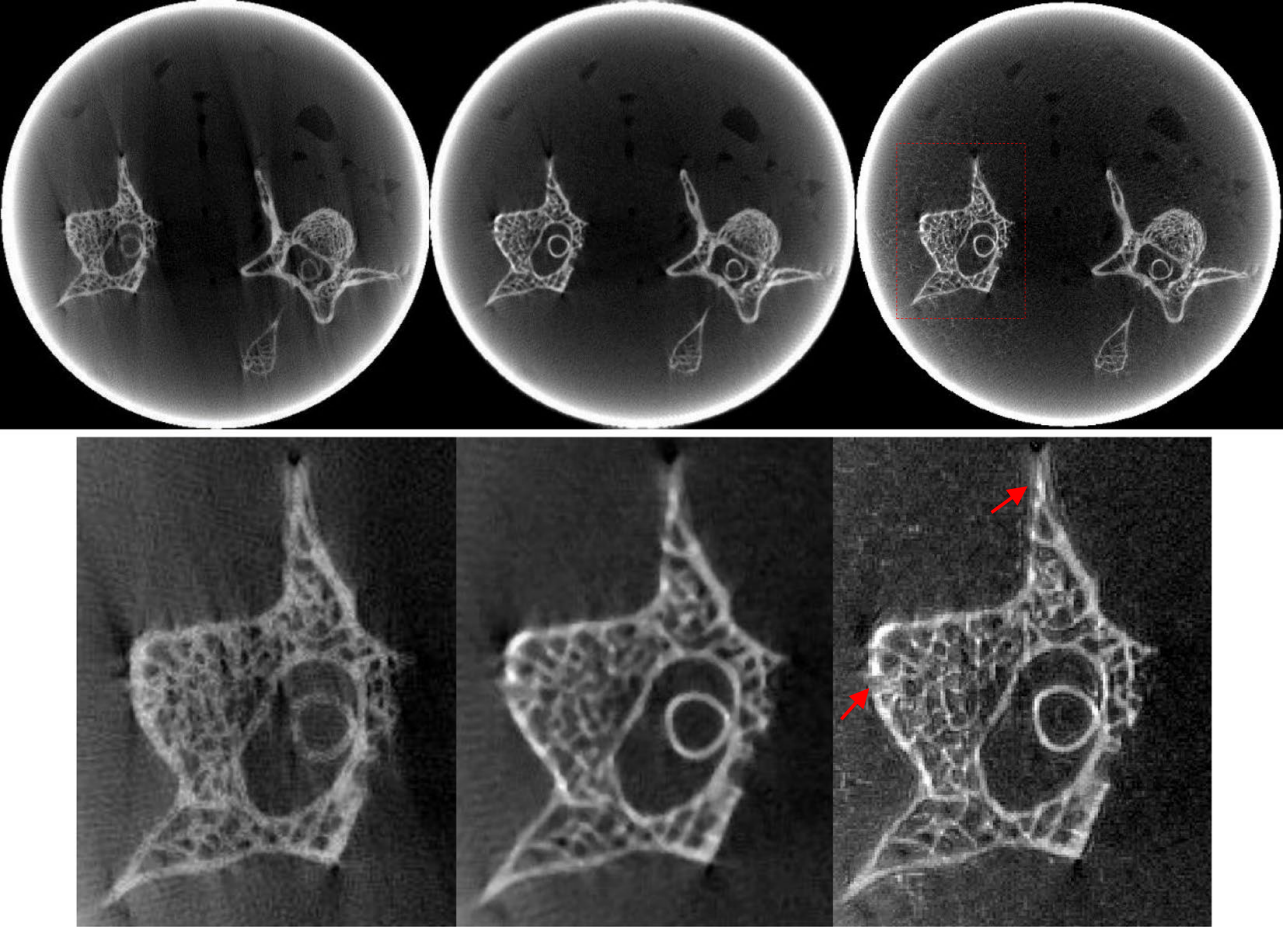
The mouse bone sample data collected over a 197.88° range reconstructions by different methods. From left to right, the images of the first row are the results obtained using FBP, PWLS-TV and PWLS-TV-FR method. The images of the second row are the partial enlarged view of the first row.

Figures 8 and 9 show images and zoomed images reconstructed using FBP, PWLS-TV and PWLS-TV-FR. Figure 8 is a reconstruction using sparse-view sampling of the anesthetized mouse projection data with 225 views, which shows that the PWLS-TV-FR method can suppress severe artifacts and preserve fine tissue structure from sparse-view sampling data in the clinic. Figure 9 is the reconstruction using limited-angle sampling data of mouse bone sample projection data using a scanning angle of 197.88°. Image reconstructions using FBP showed severe streak artifacts resulting in low detail visibility. The images reconstructed using the other two algorithms show a lower level of streak artifacts. However, the features reconstructed using PWLS-TV-FR are clearer than those reconstructed by PWLS-TV. In conclusion, the experimental micro-CT data demonstrate that the presented method can recover structural information lost using the PWLS-TV method for sparse-view or limited-angle sampling data.

## Conclusion and Discussion

To address the global use of high-diagnostic CT images obtained from sparse-view or limited-angle projection data, we improved on the statistical iterative algorithm by including an FR step. Two key components were included in this algorithm. One is a TV minimization model using PWLS criteria that account for the statistical nature of projections. The other is an FR step that is performed after each iteration of PWLS-TV to recover any missing fine features. The improved method is designed to relieve the piecewise constant assumption of TV-based methods for sparse-view or limited-angle CT image reconstruction. The reconstruction results from a digital phantom, anesthetized mouse and a mouse bone sample dataset show that the proposed PWLS-TV-FR approach is a significant improvement over the PWLS-TV method in terms of the preservation of structural details and the suppression of undesired patchy artifacts. This result demonstrates its potential to reduce CT radiation doses by decreasing the number of projection views or the rotation angle of the scan.

An iterative reconstruction algorithm usually involves numerous parameters, which can significantly impact reconstruction properties. In this work, we try our best to select the “optimal” parameters for different algorithms in order to make a fair comparison on their performance. For example, the smoothing parameter *β* balancing the degree of agreement between the estimated and the measured data in function (2) should be a small value to avoid data distortion, however, after testing, we found that ${\rm{\beta }}={e}^{-2}$ is better. Furthermore, the image patch size in the feature descriptor designed to distinguish structure from noise and artifacts has to be carefully selected because the large one leads to better detail-detection ability, while the small one can benefit the computational load. According our previous study, the patch size of $3\times 3$ is a good choice for balancing the structure detection ability and computational efficiency. Additionally, the parameter C is introduced for numerical stability to avoid the condition that ${\sigma }_{p}^{2}+{\sigma }_{q}^{2}$ is very close to zero. Therefore, it is assigned $1.25\times {10}^{-5}$ as a small constant in our work.
